# Houses and Slums

**Published:** 1924-03

**Authors:** 


THE
### HOSPITAL and HEALTH REVIEW

### HOUSES AND SLUMS.

That the new Government should place housing in the forefront of their domestic policy was inevitable. It is probable that for several years to come every fresh Administration will have to do likewise, since it is vain to suppose that the existing lack of dwelling-houses will quickly be made good. There is no royal road to the filling of the gaps that have been accumulating for thirteen or fourteen years. It is essential that it should be understood that had there been no war there would still have been a housing question. It would not have been so urgent or so difficult a question, but it would have been serious nevertheless. The unhappy Finance Act of 1910, which, after it had accomplished a great deal of mischief, had to be ignominiously repealed, made it no longer worth while to build houses, and began to create a scarcity so soon as the dwellings that were standing empty were absorbed. That was the genesis of the first Rent Restrictions Act, which has landed the country in another crop of difficulties. Then came the long war, when neither men, nor money, nor materials were available for building; and when it was over costs were almost prohibitive. This series of disasters has produced a scarcity of houses which, in a less dumbly patient land, might have produced social disorders not to be remedied by bricks and mortar. Thus it comes about that the building of new houses and the removal of old and rotten ones have brought about a domestic difficulty which no money, no energy, no enterprise can remove quickly. Those things can help enormously, but at the best much time will be needed to enable supply to keep pace with demand.

That Mr. MacDonald recognises the force of these facts there is every reason to believe. His statement on the subject to the House of Commons contained no trace of the wild talk to which some of his followers have accustomed us. To demand "millions of houses" in the twinkling of an eye is to cry for the moon. If thousands of millions of money were available for the purpose to-morrow the thing could not be done. Men are as essential as money, and men at present are not forthcoming. It is true that the Prime Minister is sanguine of being able, very soon, to arrange with the building trade unions a scheme of dilution which will do something towards reducing the scarcity of artisans which has been brought about by very much the same causes as the scarcity of houses. He is confident of success in this direction, and should he achieve it he will make good his title to be a diplomatist of the first water. A good understanding with the unions is a necessary preliminary to any comprehensive building scheme that can be devised, and the Government's scheme is still "*à l'étude.*" There is no ground for complaint in that, since no Administration which has been in office a very few weeks can reasonably be expected to have elaborated a cut-and-dried plan for the cure of an ill which goes so deep. About the nature of that plan the Government themselves are still probably very much in the dark. Mr. MacDonald would not commit himself beyond the announcement that they intend to get houses built for £500 which can be let at nine shillings a week, including rates. He recognises that this can only be done by the continuance of subsidies; but mere continuance will clearly be insufficient. A substantial increase will be imperative, and it may easily be found that the subsidy will need to be doubled. This would mean a heavy burden upon both rates and taxes, more especially if the rate of building were much expedited. The Prime Minister's sense of responsibility led him to admit the difficulty of the situation, and even that the Government's ideas may need revision. Happily Mr. Neville Chamberlain's Act, which is working so successfully, is not to be interfered with, although Mr. MacDonald rather oddly imagines that it "is going to be fruitful, if it is fruitful at all" in houses for sale. Of its fruitfulness, in view of the fact that 98,000 houses have now been authorised under it, we should have supposed that there could be no question.

When the Government's plans are complete and have been presented to the country there will be every desire to receive them with favour, provided that they steer clear of extravagances and do not seek to place an unjustifiable burden upon the country. There will be the greater readiness to help in solving the housing difficulty, because the nation is growing increasingly conscious of the pressure of another and consequential subject—the scandal of the slums. That scandal cannot be seriously attacked until the scarcity of houses has been greatly reduced. At present not only are the unhappy dwellers in the slums without hope, but their number is ever increasing because slums are ever growing. The extent of that growth, so far as London is concerned, has just been indicated by Lieut.-Colonel Levita, the Chairman of the Housing Committee of the London County Council, in a lecture at the Society of Arts. The Council has in hand the reconstruction of seven insanitary areas at a cost of a million; but there are nearly a thousand more, involving a hundred thousand people, waiting to be reconstructed, more than half of which will "brook no delay." It is impossible to accomplish so huge a task in any reasonable amount of time without heroic measures, and heroic measures are also impossible in existing circumstances. Thus it once more becomes clear that housing is a necessary preliminary to slum clearance—all along the line the one waits upon the other, for this is no mere London question. The whole of the country is involved in it, and although the difficulty may be more acute in London than elsewhere, it is very serious in almost every great town. And even when the housing scarcity has been remedied we shall have to face problems which the mere liberation of labour and materials will not solve. Sums of money will have to be provided so vast that neither the Exchequer nor the municipalities will be able to find them unless their expenditure is spread over a long period. But the country cannot afford these long delays, and a large Housing Loan may yet be necessary. We cannot continue to poison one generation after another by cooping up hundreds of thousands of people in noisome corners where the average of both physical and spiritual vitality is low and the seeds are sown of bodily decay and moral degeneracy.

